# Different nitrogen uptake patterns of plant and soil microorganisms in the forest-grassland transition zone on the Loess Plateau

**DOI:** 10.3389/fpls.2024.1480517

**Published:** 2025-01-21

**Authors:** Lina Wang, Xu Deng, Ying Zhou, Xueqi Geng, Zeling Zhang, Yakun Tang

**Affiliations:** ^1^ College of Forestry, Northwest A&F University, Yangling, Shaanxi, China; ^2^ State Key Laboratory of Soil Erosion and Dryland Farming on the Loess Plateau, Northwest A&F University, Yangling, Shaanxi, China

**Keywords:** forest–grassland transition zone, microorganisms, niche complementarity, ^15^N tracer, nitrogen uptake, plants

## Abstract

**Introduction:**

It is unclear whether plants and microorganisms achieve niche complementarity by taking up different inorganic nitrogen (N) forms to alleviate N competition, particularly in N–limited regions.

**Methods:**

This paper conducted a 15-day ^15^N tracer study (^15^NH_4_NO_3_ or ^15^NH_4_NO_3_) *in situ* to quantitatively calculate the uptake rates of plants and microorganisms in four stands (pure *Hippophae rhamnoides* L, pure *Pinus tabuliformis* Carrière, mixed *H. rhamnoides*–*P. tabuliformis*, and *Artemisia gmelinii* Weber ex Stechm grassland) in the forest–grassland transition zone on the Loess Plateau during the growing season. Among them, *H. rhamnoides* and *P. tabuliformis* can associated with arbuscular mycorrhizal and ectomycorrhizal, respectively.

**Results:**

The results indicated that *H. rhamnoides* in the pure stand and *A. gmelinii* preferred to take up ^15^NO_3_^–^, whereas *P. tabuliformis* in the pure stand preferred ^15^NH_4_^+^. Compared to pure stands, mixed afforestation decreased the NH_4_^+^ and NO_3_^–^ uptake rate of *H. rhamnoides* by 87% and 70%, respectively, but did not alter the N preference of plants. Plants and microorganisms differed in their N preferences in the pure stand, whereas this was not the case in the mixed stand. The proportional similarity index between *H. rhamnoides* and *P. tabuliformis* (0.90 ± 0.01) was higher than that between plants and microorganisms in forest stands, except for *P. tabuliformis* and microorganisms in the mixed stand (0.90 ± 0.02).

**Discussion:**

Those results indicated that niche complementarity by preferring different N forms can alleviate N competition. This study helped to gain a deeper understanding of the plasticity of N uptake patterns by plants and microorganisms in the forest–grassland transition zone, and provides theoretical support for vegetation restoration during the implementation of the Grain for Green program on the Loess Plateau.

## 1 Introduction

Soil inorganic nitrogen (N), NH_4_^+^ and NO_3_^–^, is the primary N source for the majority of plants and is often considered a limiting factor for ecosystem productivity (Andersen and Turner, 2013; Cui et al., 2017; Zhou et al., 2020; He et al., 2022). Previous studies demonstrated that N demand may lead to competition for NH_4_^+^ and NO_3_^–^ among different plants and between plants and microorganisms under N limiting conditions (Kuzyakov and Xu, 2013; Song et al., 2017; Hu et al., 2021). Coexisting species and microorganisms take up different N forms, i. e. niche complementarity, which has been considered the main mechanism of alleviating soil N limitation (Miller et al., 2007; Ashton et al., 2010; Liu et al., 2020). Human activities have significantly affected the chemical forms of atmospheric inorganic N deposition over recent decades (Fowler et al., 2013; Ackerman et al., 2018; Yu et al., 2019; Gurmesa et al., 2022). Therefore, understanding the inorganic N uptake patterns by plants and microorganisms is essential for optimizing resource utilization in N–limited ecosystems.

Soil N availability was acknowledged to influence N uptake by plants (Zhu et al., 2019; Hu et al., 2021). In general, plants prefer the most abundant form of N in the soil (Mckane et al., 2002; Kahmen et al., 2006; Cui et al., 2017; Liu et al., 2020). In addition, different mycorrhizal symbiosis types, such as arbuscular mycorrhizal (ACM) and ectomycorrhizal (ECM), have also been suggested as important factors affecting N uptake of plants (Liu et al., 2020). ACM increases N uptake by decomposing organic matter and generally acquires high mobility of NO_3_^–^ faster than other mycorrhizal types (Xie et al., 2022). Conversely, plants associated with ECM take up more NH_4_^+^ compared to NO_3_^–^ due to the high assimilation of NH_4_^+^ by extraradical mycelium (Javelle et al., 2008; Phillips et al., 2013; Zhang et al., 2019). However, it is still unclear whether the plant N preferences are influenced by different root associations with mycorrhizal types under similar soil N availabilities.

Competition among plants and between plants and microorganisms in N–limited regions have been reported to exert impacts on plant N uptake rate and preference (Kahmen et al., 2006; Andersen et al., 2017; Hu et al., 2021). Liu et al. (2021) demonstrated that plants dependent on ACM (*Michelia maudiae* and *Schima superba*) exhibit inhibitory effects on inorganic N uptake of two ECM plants (*Pinus massoniana* and *Pinus elliottii*) in mixed forests. In addition, microorganisms are often considered the main competitors of plants for soil N, as they grow rapidly and have a high surface area–to–volume ratio (Hodge et al., 2000; Harrison et al., 2007; Andersen et al., 2017). Traditionally, it was assumed that microorganisms are more inclined to prefer NH_4_^+^ due to its lower energy costs (Fraterrigo et al., 2011; Wang and Macko, 2011; Liu et al., 2016). However, some experiments indicated that microorganisms can take up NO_3_^–^ as efficiently as NH_4_^+^ (Inselsbacher et al., 2009; Kaštovská and Šantrůčková, 2011), presumably due to the higher NO_3_^–^ mobility in soils (Hodge et al., 2000; Song et al., 2007; Inselsbacher et al., 2009). Currently, most studies use roots or seedlings in hydroponic and pot experiments to investigate plant N preferences (Templer and Dawson, 2004; Li et al., 2016; He et al., 2022). The results, however, could not take into account the impact of N competition among plants and between plants and microorganisms on the N uptake of plants (Uscola et al., 2017; Zhou et al., 2020). Therefore, quantifying the degree of competition among plants and between plants and microorganisms is recommended in characterizing plant N uptake patterns in N–limited conditions.

Severe soil erosion on the Loess Plateau has led to a lack of soil N in the region (Chen et al., 2021; Yan et al., 2022; Tang et al., 2024). To enhance soil and water conservation, policies related to “Grain for Green” have been implemented (Fu et al., 2016; Tang et al., 2019). However, the lack of N during vegetation reconstruction also poses a challenge to plant growth (Gao et al., 2018). Yu et al. (2019) found that the amount of inorganic N deposited has increased, and NHx was slightly higher than NOy over the past decade on the Loess Plateau. Studies have revealed that *Hippophae rhamnoides* L and *Pinus tabuliformis* Carrière can associate with ACM and ECM, respectively (Zhang et al., 2017; Tang et al., 2019), which are ecological and economic plants in the region (Wang et al., 2022; Ning et al., 2023). *Artemisia gmelinii* Weber ex Stechm is a typical herbaceous plant species in this forest–grassland transition zone with a wide coverage area (Li et al., 2022). Additionally, mixed plantations of *H. rhamnoides* with *P. tabuliformis* have been widely used on the Loess Plateau due to the high soil conservation capacity (Tang et al., 2019). Therefore, understanding the N uptake patterns of plants and microorganisms is crucial to improving the available N utilization efficiency under soil N–limited conditions on the Loess Plateau.

This study conducted *in situ* ^15^N labeling experiments (NH_4_^+^ and NO_3_^–^) in four stands (*H. rhamnoides*, *P. tabuliformis*, *H. rhamnoides*–*P. tabuliformis* mixed stand, and *A. gmelinii*) in the forest–grassland transition zone of the Loess Plateau. The main intentions of this research include: (1) examining the NH_4_^+^ or NO_3_^–^ preferences of *H. rhamnoides*, *P. tabuliformis*, and *A. gmelinii*; and (2) assessing the degree of interspecific plant competition and competition between plants and microorganisms for soil N in different stands. Based on previous studies, the following hypotheses were made: (1) *H. rhamnoides* and *P. tabulaeformis* maytake up NO_3_^–^ and NH_4_^+^, respectively, due to differences in the association of their roots with mycorrhizal types; (2) *A. gmelinii* prefer to take up the dominant N forms in the soil; (3) microorganisms primarily take up NH_4_^+^ with low energy efficiency.

## 2 Materials and methods

### 2.1 Study area

This research was performed in Ansai ecological station of the Chinese Academy of Sciences (109.3137°E, 36.8723°N, 1230–1237 m above sea level, **Supplementary Figure S1**), in the forest–grassland transition zone on the Loess Plateau. The area with a temperate semi–arid climate (Tang et al., 2018a). The average annual air temperature is 10.7 ± 0.1°C and the average annual precipitation is 456.9 ± 5.7 mm (2000–2021, mean ± SE). Three forest stands planted in 2004 and one naturally growing grassland were selected as the study sites, i.e., pure *H. rhamnoides* stand, pure *P. tabuliformis* stand, mixed *H. rhamnoides*–*P. tabuliformis* stand, and *A. gmelinii* stand. The soil in this area was divided into silt loam soil depending on the USDA soil taxonomy classification with 65% silt, 22% sand, and 13% clay (Tang et al., 2024). The detailed data of soil has been shown in **Table 1**. According to Güsewell (2004) and Sorrell et al. (2011) description, the N: P ratio of plant leaves in this area ranges from 11.26 (*P. tabuliformis* (p)) to 15.65 (*H. rhamnoides* (p)) (**Table 2**), which belongs to the N-limited zone. Three replicated plots were selected for each forest (20 m × 20 m) and grassland (10 m × 10 m) stands. The understory grasses were *Glycyrrhiza uralensis* and *Bothriochloa ischaemum* in forest stands (Tang et al., 2018b). The original tree density for *H. rhamnoides* and *P. tabuliformis* in the pure stands (*H. rhamnoides* (p) and *P. tabuliformis* (p), respectively) were 3025 trees ha^–1^, with a spacing of 2 m between each plant. The *H. rhamnoides* (1650 trees ha^–1^) and *P. tabuliformis* (1375 trees ha^–1^) in the mixed stand (*H. rhamnoides* (m) and *P. tabuliformis* (m), respectively) have six and five rows, respectively. *P. tabuliformis* were planted in a 4 m gap between rows of *H. rhamnoides*. The vegetation coverage rates were 80.72%, 74.76%, 87.01%, and 85.64% in the *H. rhamnoides* pure stand, *P. tabuliformis* pure stand, mixed stand, and grassland, respectively. The coverage rates of *H. rhamnoides* and *P. tabuliformis* in the mixed stand were 47.43% and 63.30%, respectively. Tree crown size of *H. rhamnoides* (p), *P. tabuliformis* (p), *H. rhamnoides* (m) and *P. tabuliformis* (m) were 2.18 m^2^, 4.22 m^2^, 1.92 m^2^, and 4.73 m^2^ (**Table 2**).

**Table 1:** Soil properties in the 0–50 cm soil for the four studied stands.

Sample stand	TN (g kg^–1^)	TC (g kg^–1^)	TP (g kg^–1^)	AP (mg kg^–1^)	C: N	NH_4_^+^ concentration (mg kg^–1^)	NO_3_^–^ concentration (mg kg^–1^)	NH_4_^+^:NO_3_^–^	pH	Moisture (%)	*R_mineralization_* (mg kg^–1^ d^–1^)	*R_nitrification_* (mg kg^–1^ d^–1^)	*R_ammonification_* (mg kg^–1^ d^–1^)
*H. rhamnoides* pure stand	0.35 (0.02)^a^	19.91 (0.12)^a^	0.54 (0.01)	1.38 (0.35)	58.46 (2.32)	2.37 (0.32)^a^	1.17 (0.20)^a^	2.02 (0.20)^b^	8.86 (0.02)	15.05 (0.60)	0.51 (0.22)^a^	0.49 (0.18)^a^	0.03 (0.05)
*P. tabuliformis* pure stand	0.31 (0.01)^a^	19.21 (0.19)^b^	0.53 (0.02)	1.28 (0.13)	63.48 (1.90)	2.15 (0.20)^ab^	0.67 (0.08)^b^	3.21 (0.48)^a^	8.90 (0.02)	15.48 (0.52)	0.20 (0.08)^a^	0.15 (0.04)^ab^	0.05 (0.05)
Mixed stand	0.31 (0.01)^a^	18.90 (0.23)^b^	0.55 (0.01)	1.35 (0.27)	63.65 (2.25)	1.71 (0.14)^b^	0.78 (0.11)^b^	2.19 (0.23)^b^	8.84 (0.04)	15.01 (0.58)	0.09 (0.07)^b^	0.08 (0.04)^b^	0.01 (0.05)
Grassland	0.25 (0.01)^b^	17.59 (0.22)^c^	0.53 (0.01)	1.34 (0.23)	71.23 (0.83)	1.91 (0.18)^ab^	0.67 (0.05)^b^	2.85 (0.17)^ab^	8.94 (0.02)	14.22 (0.60)	0.09 (0.14)^b^	0.13 (0.09)^b^	–0.04 (0.09)

Each value is presented as mean value with SE in parentheses. Different superscript letters indicate significant differences among four stands (*P*< 0.05).

**Table 2:** Plant properties in the four studied stands.

Plant	Tree type	Height (m)	Diameter (cm)	Crown diameter of north–south × east–west (m)	Leave N concentrations (%)	Leave P concentrations (%)	Leave N:P	Mycorrhizal type	Colonization rate (%)	NR (U L^–1^)	GS (U L^–1^)
*H. rhamnoides* (p)	Deciduous broadleaf	4.01 (0.11)	5.13 (0.14)	1.58 (0.07)×1.53 (0.07)	3.22 (0.03)^a^	22.26 (0.61)^ab^	15.65 (0.43)	ACM	52.92 (3.94)	43.81 (2.92)^b^	18.90 (2.85)^c^
*P. tabuliformis* (p)	Evergreen coniferous leaves	4.24 (0.07)	7.09 (0.12)	2.24 (0.13)×2.30 (0.14)	0.93 (0.04)^d^	10.63 (0.26)^b^	11.26 (0.14)	ECM	50.26 (2.63)	51.67 (3.00)^ab^	26.66 (2.98)^b^
*H. rhamnoides* (m)	Deciduous broadleaf	3.71 (0.07)	4.79 (0.12)	1.6 (0.06)×1.53 (0.06)	3.24 (0.05)^a^	23.00 (0.36)^ab^	14.91 (0.26)	ACM	49.69 (4.93)	58.04 (4.31)^a^	34.23 (1.60)^a^
*P. tabuliformis* (m)	Evergreen coniferous leaves	4.05 (0.09)	6.51 (0.14)	2.42 (0.07)×2.49 (0.09)	1.33 (0.03)^c^	15.24 (0.74)^ab^	12.85 (0.42)	ECM	43.31 (3.28)	53.11 (3.18)^ab^	31.40 (1.62)^ab^
*A. gmelinii*		0.33 (0.13)	0.01 (0.00)	0.06 (0.01)×0.13 (0.03)	1.82 (0.02)^b^	27.30 (1.02)^a^	11.95 (0.51)			53.70 (6.63)^ab^	31.12 (3.14)^ab^

Each value is presented as mean value with SE in parentheses. Different superscript letters indicate significant differences between the four stands (*P*< 0.05). NR, nitrate reductase activity; GS, glutamine synthetase activity. (p) and (m) represent pure and mixed stands, respectively.

### 2.2 Experimental design

The experiment was set up in June and August 2022. To analyze the inorganic N uptake patterns of plants and microorganisms, there were two types of ^15^N labeling solutions, ^15^NH_4_NO_3_ (99.12 atom%) and NH_4_^15^NO_3_ (99.21 atom%) were employed. A solution with the same amount of unlabeled NH_4_NO_3_ was used as the control (Kaštovská and Šantrůčková, 2011). To avoid N fertilization effects, ^15^N was added at a low rate of 0.441 g m^–2^, which was equal to 8% of local atmospheric N deposition in the study region (20 kg N ha^–1^ year^–1^) (Guo et al., 2020). Adjacent *H. rhamnoides* and *P. tabuliformis* received the same labeling solutions simultaneously in the mixed stand. Therefore, each plant can accept the same molar concentration of NH_4_^+^–N and NO_3_^–^–N. The total amount of added N did not cause additional disturbance of the different N pools in the soil (including total N, NH_4_^+^, and NO_3_^–^; P > 0.05) (**Supplementary Table S1**). In addition, ceramic soil suction samplers were installed in each plot to collect soil solution at a depth of 80 cm. A subplot (2 m × 2 m) was randomly selected within each plot to collect surface runoff.

The labeling experiment was conducted on 25 July 2022. Three subplots of 2.5 m × 2.5 m and 1 m × 1 m were established in each of the forest and grassland plots to receive three N forms. Three replicates per treatment were established in each plot. To exclude competition effects on N uptake patterns from other plants, the grasses in each forest plot were cut manually by clipping stems 10 cm above the ground (Zhu et al., 2021). In each forest and grassland subplot, the tracers were dissolved in 31.25 L and 5 L of pure water (approximately equal to 5 mm precipitation), respectively, which was approximately 1% of the local rainfall. Two backpack sprayers were used to spray the solution evenly and directly on the soil surface to avoid cross contamination (Zhou et al., 2020). The litter in the sample subplot was removed before ^15^N labeling to prevent the litter retaining ^15^N tracers and then returned to its original location after ^15^N labeling. Several rainfall events occurred after ^15^N labeling, with daily precipitation ranging from 0.0 to 24.2 mm (**Supplementary Figure S2**), which promoted infiltration of the ^15^N tracer. Furthermore, leachate in the 80 cm soil layer and lateral surface runoff were not observed at any sampling times. This result indicated that most of the labeled solution was retained in the 0–50cm soil layer. If the δ^15^N by plant organs and whole plant in the NO_3_^–^ labeled treatment were more than in the NH_4_^+^ labeled treatment, then the plants preferred NO_3_^–^, and vice versa (Wang and Macko, 2011; Zhou et al., 2020).

The plant organs and soil samples in each subplot were collected at 3 (28 July), 7 (1 August), and 15 days (9 August) after ^15^N tracer application. The organs of trees were divided into leaves, branches, and roots. The organs of grass were divided into leaves and root. Fine roots (< 2 mm) were manually selected from the soil sample. To determine mycorrhizal colonization rates, additional fine roots were collected from each of the four cardinal directions (north, south, east, west) from trees in the control subplot before labeling (n = 9). Roots were then extracted from blocks by washing with water and maintained at 4 °C. The soil samples were collected using a 6 cm diameter spiral drill and divided into two layers (0–20 and 20–50 cm). More than 75% of the fine roots were concentrated within those layers (**Supplementary Figure S3**). All samples from each subplot were pooled into one composite sample. The soil sample was divided into two portions. One portion was stored at −20°C until subsequent microbial determination. The other portion was used to determine the soil properties, including total carbon content (TC), total N content (TN), total phosphorus (TP), available phosphorus (AP), pH, and the concentration of NO_3_^–^ and NH_4_^+^. Plant samples were also transferred to the laboratory for determination of the total N concentrations and δ^15^N. An *in situ* soil core cultivation method was used to determine the net N conversion rate (Xu et al., 2007). Two polyvinyl chlorid cylinders were driven into the soil on each of 3, 7, and 15 days after labeling. The soil in one of the polyvinyl chlorid tubes was extracted to determine the initial concentrations of inorganic N, while the other was incubated in the soil for 15 days before determining inorganic N.

### 2.3 Chemical analyses

All plant leaves, branches, and root samples were cleaned using deionized water and then dried to a constant weight at 75°C. Soil sample, after air drying, was sieved through a 2 mm sieve and then maintained at 4°C until measurements. Plants and soil samples were ball–milled and the TC, TN, and δ^15^N were analyzed by elemental analyzer–isotope ratio mass spectrometry (Finnigan MAT253, Thermo Finnigan, Germany). TP and AP concentration of the soil were analyzed by the HClO_4_–H_2_SO_4_ colorimetric (Wang et al., 2012). The concentration of inorganic N, extracted by 2 mol L^–1^ potassium chloride, was measured using a high–performance flow injection auto–analyzer (AA3, SEAL, Germany) (Zhou et al., 2020). A pH meter (Sartorius PB-10, Gottingen, Germany) was used to gauge soil pH in 1:2.5 (soil/solution ratio) water extract. To measure the soil moisture, partial soil samples were dried at 105°C for 2 d. Microbial biomass N and δ^15^N were determined using the chloroform fumigation–extraction technique according to Andersen et al. (2017) and Ma et al. (2021). Briefly, a portion of fresh soil was treated with chloroform fumigation for 24 h, and the fumigated sample and another portion of fresh soil were removed using 0.5 mol L^–1^ potassium sulfate. Subsequently, the solutions were filtered, freeze–dried, and analyzed for N and δ^15^N concentrations. The enzyme–linked immunosorbent assay kit was used to determine the activities of plant root nitrate reductase (NR) and glutamine synthetase (GS) (JLC455621 and JLC455326, Jining Industrial Co., Ltd, Shanghai) (Ji et al., 2024). The 5% acetic acid ink and 0.05% trypan blue methods were used to detect the ACM and ECM infection rates of plant roots, respectively (Phillips and Hayman, 1970; Vierheilig et al., 1998).

The ^15^N uptake rate (µg ^15^N g^–1^ d^–1^) was estimated using the following formulas (Templer and Dawson, 2004):


(1) $${\;}^{15}N\;uptake=\frac{N\;concentrations\times \frac{APE}{100}}{time\times \frac{atom{\%}^{15}Ntracer}{100}}$$

(2) $$APE=atom{\%}_{labeled}-atom{\%}_{control}$$

where *APE* represents the atom% excess; *atom%_labeled_* and *atom%_control_* represent the atom% in the labeled and unlabeled organs, respectively; *N concentrations* represent the dry masses of N in the N pool (leaves, branches, roots, or microorganisms, µg g^–1^); and *atom%^15^N tracer* is the atomic percentage of applied ^15^N–labeled N (99.12 atom% for ^15^NH_4_NO_3_ and 99.21 atom% for NH_4_^15^NO_3_). The N uptake rate of whole plants was calculated by summing the weighs of the N concentrations of the different organs.

The net mineralization (*R_mineralization_*), nitrification (*R_nitrification_*), and ammonification (*R_ammonification_*) rates were calculated using the following formulas (Xu et al., 2007):


(3) $$R_{\min eralization}=\frac{c{\left(NH_{4}^{+}+NO_{3}^{\_}\right)}_{t2}-c{\left(NH_{4}^{+}+NO_{3}^{\_}\right)}_{t1}}{15}$$

(4) $$R_{nitrification}=\frac{c{\left(NO_{3}^{\_}\right)}_{t2}-c{\left(NO_{3}^{\_}\right)}_{t1}}{15}$$

(5) $$R_{ammonification}=\frac{c{\left(NH_{4}^{+}\right)}_{t2}-c{\left(NH_{4}^{+}\right)}_{t1}}{15}$$

where *t1* and *t2* represent the initial and after incubation time, respectively; *c* is the mean N concentration.

The proportional similarity (*PS*) index was used to evaluate the degree of N competition between plants and microorganisms in the four stands, and between plants in the mixed stand. *PS* was calculated as follows (Hoekstra et al., 2014):


(6) $$PS=1-0.5|P_{1\text{i}}-P_{2\text{i}}|$$

where *P_1i_* and *P_2i_* represent the same form of N uptake proportion (NH_4_^+^ or NO_3_^–^) of the plants and microorganisms in each stand, respectively, or different plants in the mixed *H. rhamnoides*–*P. tabuliformis* stand. The *PS* index ranged from 0 to 1, and the niche overlap of the N uptake patterns increased as the *PS* index increased (Phillips and Gregg, 2003; Hoekstra et al., 2014).

### 2.4 Statistical analyses

To test for normality, the Kolmogorov–Smirnov test was applied, and Levene’s test was conducted to assess the homogeneity of variances before analyzing the data. If the normality assumption was not met, the data were log–transformed. However, the soil properties (TC, TN, TP, AP, pH, moisture, net N conversion rate, NH_4_^+^ concentration, NO_3_^–^ concentration, and NH_4_^+^:NO_3_^–^ ratio) among the four stands and in plant root–related variables (mycorrhizal colonization rate, NR, and GS activity) among plants were still not normally distributed after transformation. Therefore, the Kruskal–Wallis test and Dunn’s test were used to examine the differences among these parameters (Zhang et al., 2018). The ^15^N uptake rates of plants and microorganisms were analyzed using the repeated measurements method with sample time as a repeated variable, and N forms as factors (Zhang et al., 2019). If the interaction between N forms and sample time was significant, one–way analysis of variance (ANOVA) was conducted to analyze the ^15^N uptake rate differences of plants and microorganisms for a specific sampling date (at 3, 7, and 15 days after labeling) (Wang et al., 2020). The differences ^15^N uptake rates for the above–ground parts of plants and whole plants between the different plants, and for microorganisms between the four stands were analyzed using one–way ANOVA followed by least significant difference tests. The same method was performed to analyze the differences in *PS* index between different plants in the mixed stand and between plants and microorganisms among the four stands. All statistical tests were conducted using SPSS 26.0 (IBM Corp, Armonk, NY, USA). Graphs were prepared in Origin 2023b software.

## 3 Results

### 3.1 Soil and plant properties

**Figure 1:**
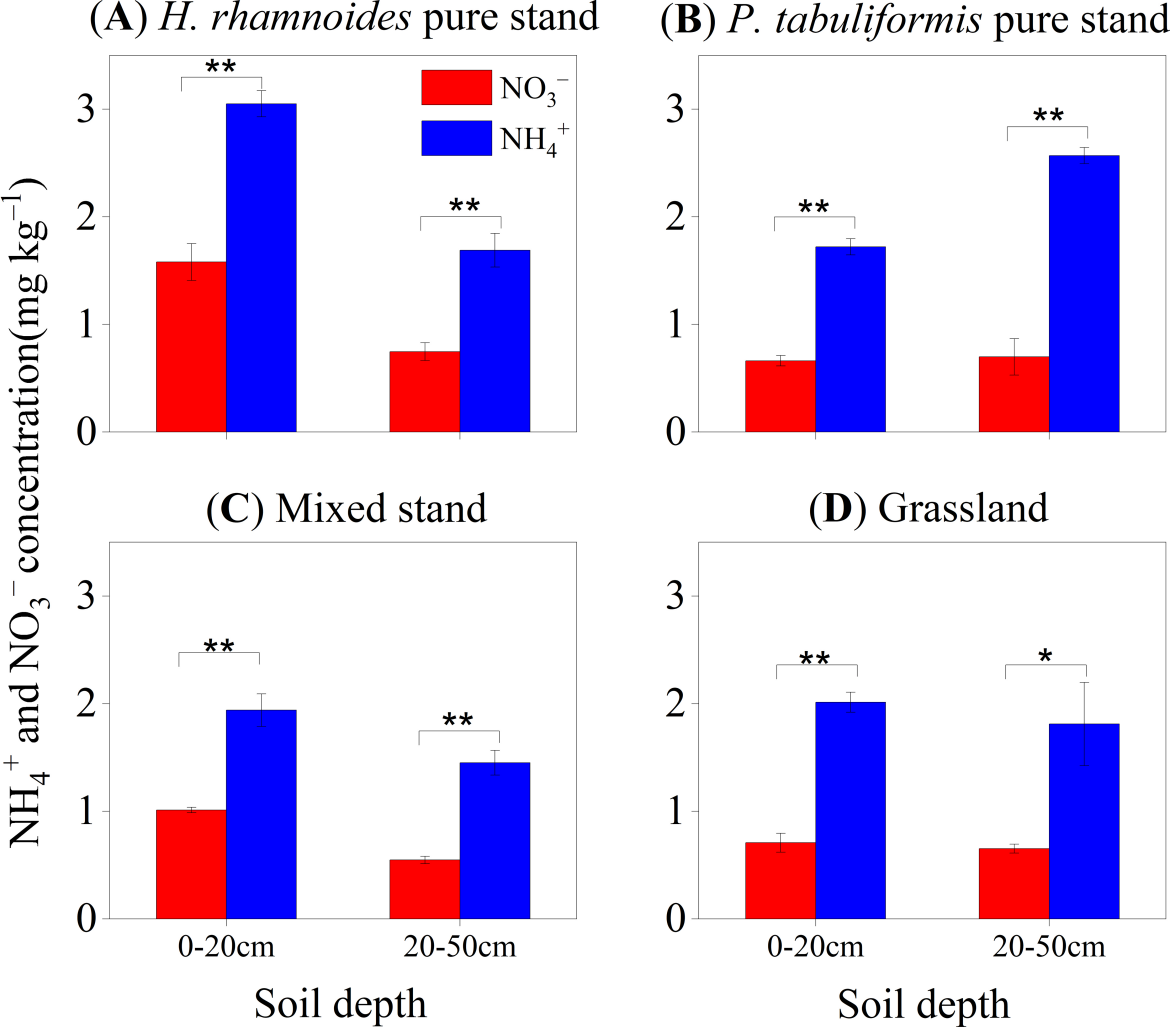
Concentrations of NH_4_^+^ and NO_3_^–^ in different soil layers in the four stands of **(A)** *H. rhamnoides*, **(B)** *P. tabuliformis*, **(C)** Mixed, and **(D)** Grassland (mean ± SE, n = 3). Error bars provide ± SE. Asterisks indicate significant differences in concentration between the two N forms in each soil layer of individual stands (*, *P*< 0.05; **, *P*< 0.01).

Ammonium was the main form of inorganic soil N in all stands (**Figure 1**), with concentrations ranging from 1.71 to 2.37 mg N kg^–1^, which was 2.02 to 3.21 times that of NO_3_^–^ in the 0–50 cm soil (**Table 1**). Furthermore, the highest inorganic N concentrations were found in the 0–20 cm soil layer of the *H. rhamnoides* pure stand (*P<* 0.05) (**Figure 1A**). In addition, the NH_4_^+^:NO_3_^–^ ratio for the *P. tabuliformis* pure stand (3.33 ± 0.48) was higher than that of the other stands. However, the *H. rhamnoides* pure stand had the highest *R_nitrification_* (0.49 ± 0.18 mg kg^–1^ d^–1^; *P*< 0.05) (**Table 1**). The soil pH was 8.86, 8.90, 8.84, and 8.94 in the *H. rhamnoides* stand, *P. tabuliformis* stand, *H. rhamnoides*–*P. tabuliformis* mixed stand, and grassland, respectively. Average soil moisture ranged from 14.22% to 15.48% and there were no significant differences in pH and soil moisture among the four stands (*P* > 0.05) (**Table 1**).

Leaf N concentrations significantly differed among the three plants. The highest and lowest leaf N concentrations occurred in *H. rhamnoides* and *P. tabuliformis* (p), respectively (*P*< 0.01) (**Table 2**). The mycorrhizal colonization rate ranged from 43.31% to 52.92%, but there was no significant difference between plants (*P* > 0.05). Moreover, there were significant differences in the NR and GS activities among the plant roots (*P*< 0.05). The range of NR and GS activity in plant roots were 43.81 U L^–1^ to 58.04 U L^–1^ and 18.90 U L^–1^ to 34.23 U L^–1^, respectively. The NR and GS activities in *H. rhamnoides* (p) were lower than those in *H. rhamnoides* (m). The roots of *H. rhamnoides* (p) also had the lowest GS activity among the three plants (*P*< 0.05) (**Table 2**).

### 3.2 Changes in δ^15^N and ^15^N uptake rates

After labeling with the ^15^N tracer, the δ^15^N in all plant organs significantly increased over the experimental period (*P*< 0.01) (**Supplementary Figures S4, S6**). Repeated measurement ANOVA showed that the δ^15^N values in plant organs with ^15^NO_3_^–^ labeling were higher than those with ^15^NH_4_^+^ labeling for *H. rhamnoides* and *A. gmelinii* (**Supplementary Figures S4A, B, S6A**). Conversely, the organs of *P. tabuliformis* exhibited higher δ^15^N values for ^15^NH_4_^+^ than for ^15^NO_3_^–^ labeling (**Supplementary Figures S4C, D**).

**Figure 2:**
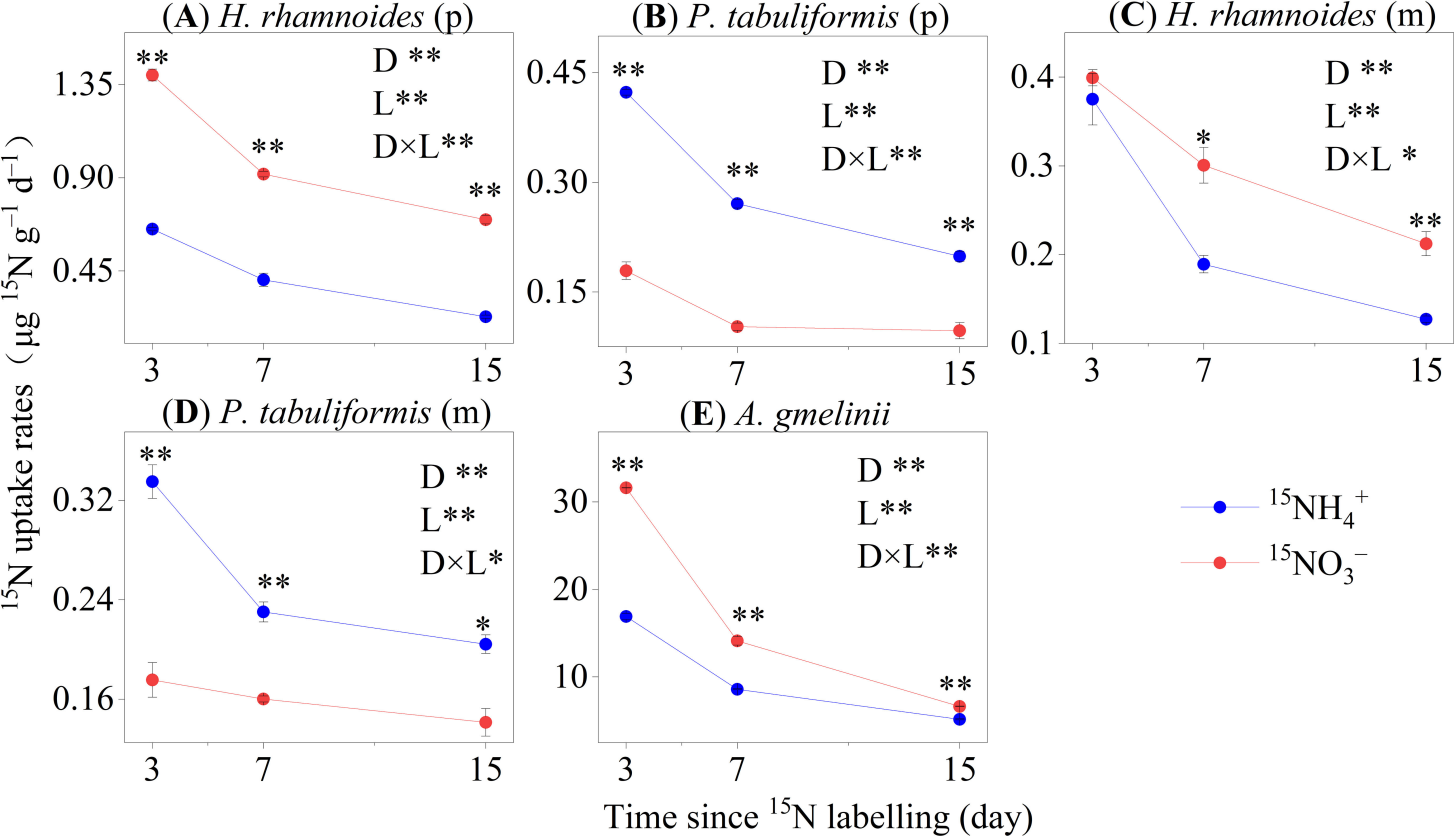
The ^15^N uptake rates of whole plants in the four studied stands of **(A, C)** *H. rhamnoides*, **(B, D)** *P. tabuliformis* and **(E)** *A. gmelinii* (means ± SE, n = 3). Error bars provide ± SE. Asterisks indicate significant differences in the ^15^N uptake rates between N forms within each stand (*, *P*< 0.05; **, *P*< 0.01). D: Time since ^15^N labeling (day); L: The form of applied ^15^N labeled N. (p) and (m) represent plants in pure and mixed stands, respectively.

The average uptake rate of ^15^NH_4_^+^ and ^15^NO_3_^–^ by trees were 0.304 ± 0.024 and 0.398 ± 0.066 µg ^15^N g^–1^ d^–1^, respectively (**Figures 2A–D**). The ^15^NO_3_^–^ uptake rates for *H. rhamnoides* (p) and *H. rhamnoides* (m) were significantly higher than those for ^15^NH_4_^+^ (*P*< 0.01). Conversely, *P. tabuliformis* had higher ^15^N uptake rates for ^15^NH_4_^+^ than for ^15^NO_3_^–^ (*P*< 0.01) in the pure and mixed stands (**Figure 2**). The *A. gmelinii* ^15^N uptake rate for ^15^NH_4_^+^ was significantly lower than that for ^15^NO_3_^–^ (*P*< 0.01) (**Figure 2E**). After ^15^N labeling, the N uptake rates of the whole plant and specific organs were significantly decreased for ^15^NH_4_^+^ and ^15^NO_3_^–^ labeled in all sample stands (**Supplementary Figures S5, S6**).

**Figure 3:**
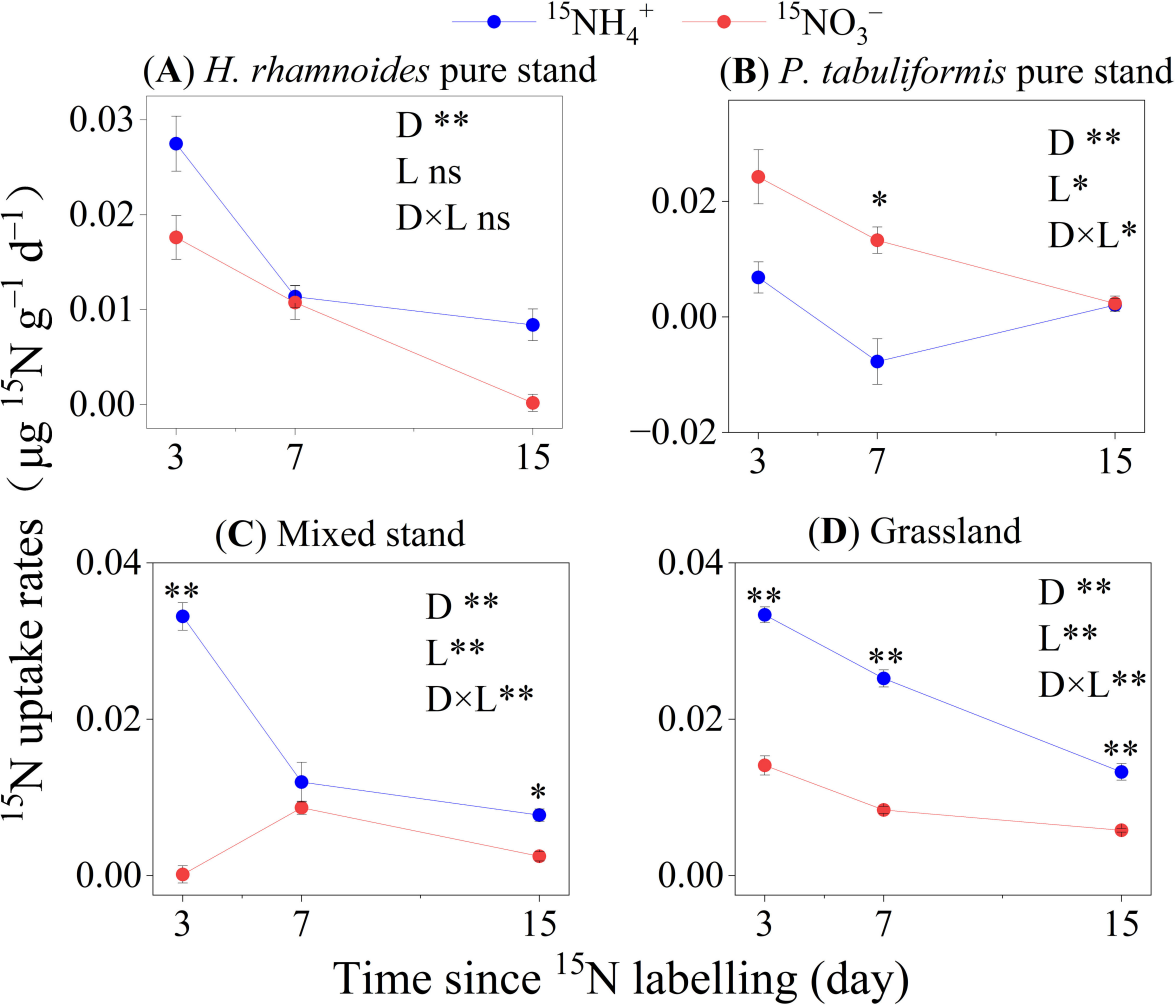
The ^15^N uptake rates of microorganisms in the four studied stands of **(A)** *H. rhamnoides*, **(B)** *P. tabuliformis*, **(C)** Mixed, and **(D)** Grassland (means ± SE, n =3). Error bars provide ± SE. Asterisks indicate significant differences in the ^15^N uptake rates between N forms within each stand (*, *P*< 0.05; **, *P*< 0.01; ns, no significant difference, p>0.05). D: Time since ^15^N labeling (day); L: The form of applied ^15^N–labeled N.

**Figure 4:**
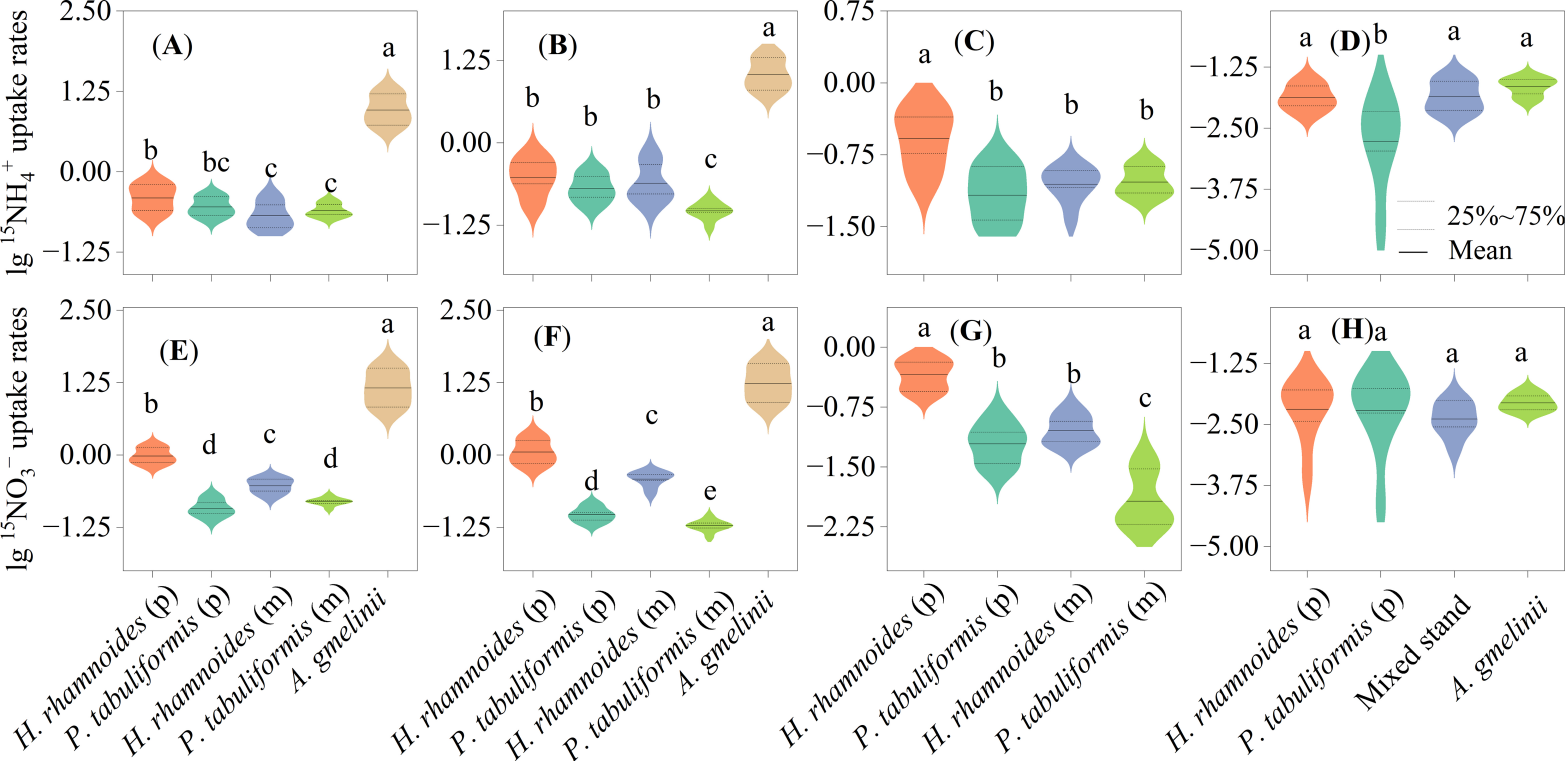
Uptake rates of ^15^N tracer of ^15^NH_4_^+^ **(A–D)** and ^15^NO_3_^–^ **(E–H)** in the whole **(A, E)**, leaves **(B, F)**, branches **(C, G)** of plants and microorganisms **(D, H)** in the four studied stands. Different letters indicate significant differences in N uptake rates between sample stands. (P) and (M) represent plants in and mixed stands, respectively.

Among the four stands, the average ^15^NH_4_^+^ and ^15^NO_3_^–^ uptake rates by microorganisms were 0.014 ± 0.002 and 0.009 ± 0.001 µg ^15^N g^–1^ d^–1^, respectively (**Figures 3A–D**). In the *H. rhamnoides* pure stand, mixed stand, and grassland, the ^15^NH_4_^+^ uptake rates for microorganisms were significantly higher than those for ^15^NO_3_^–^, while the opposite result was observed in the *P. tabuliformis* pure stand (*P*< 0.01) (**Figure 3B**). In addition, the microorganisms in the *P. tabuliformis* pure stand had the lowest ^15^NH_4_^+^ uptake rate (*P*< 0.01) (**Figures 4D, H**).

The inorganic N uptake rates of *A. gmelinii* were significantly higher than those for the two trees (*P<* 0.05) (**Figures 4A, E**). The highest N uptake rates of leaves and branches appear in *A.gmelinii* and *H. rhamnoides* (p) (P<0.01) (**Figures 4B, C, F, G**). *Pinus tabuliformis* had the lowest ^15^NO_3_^–^ uptake rate in the pure and mixed stands (*P*< 0.05) (**Figure 4E**). The ^15^NO_3_^–^ uptake rates for the three plants were in the order: *H. rhamnoides* (p) > *H. rhamnoides* (m) and *P. tabuliformis* (m) > *P. tabuliformis* (p) (*P*< 0.05) (**Figure 4E**).

### 3.3 Differences in *PS* index among the four stands

The *PS* index between *P. tabuliformis* and microorganisms in the pure stand was significantly lower than that in the mixed stand, and was the lowest among the four stands (0.74 ± 0.04; *P*< 0.05) (**Table 3**). Conversely, there was no significant difference in the *PS* index between *H. rhamnoides* and microorganisms in the pure and mixed stands. In addition, the *PS* index between *H. rhamnoides* and *P. tabuliformis* was higher than that between plants and microorganisms, except for *P. tabuliformis* and microorganisms in the mixed stand (**Table 3**).

**Table 3:** Proportional similarity (*PS*) index between plants and microorganisms in the four stands.

Stands	Object	*PS*
*H. rhamnoides* pure stand	*H. rhamnoides* and microorganisms	0.83 ± 0.03^ab^
*P. tabuliformis* pure stand	*P. tabuliformis* and microorganisms	0.74 ± 0.04^c^
Mixed stand	*H. rhamnoides* and microorganisms	0.82 ± 0.03^b^
	*P. tabuliformis* and microorganisms	0.90 ± 0.02^a^
	*H. rhamnoides* and *P. tabuliformis*	0.90 ± 0.01^a^
	Plants and microorganisms	0.85 ± 0.03 ^ab^
Grassland	*A. gmelinii* and microorganisms	0.84 ± 0.01^ab^

Each value is presented as mean value with SE in parentheses. Different superscript letters indicate significant differences of *PS* index among the four stands (*P*< 0.05).

## 4 Discussion

### 4.1 Uptake of different N forms by the three plants

**Figure 5:**
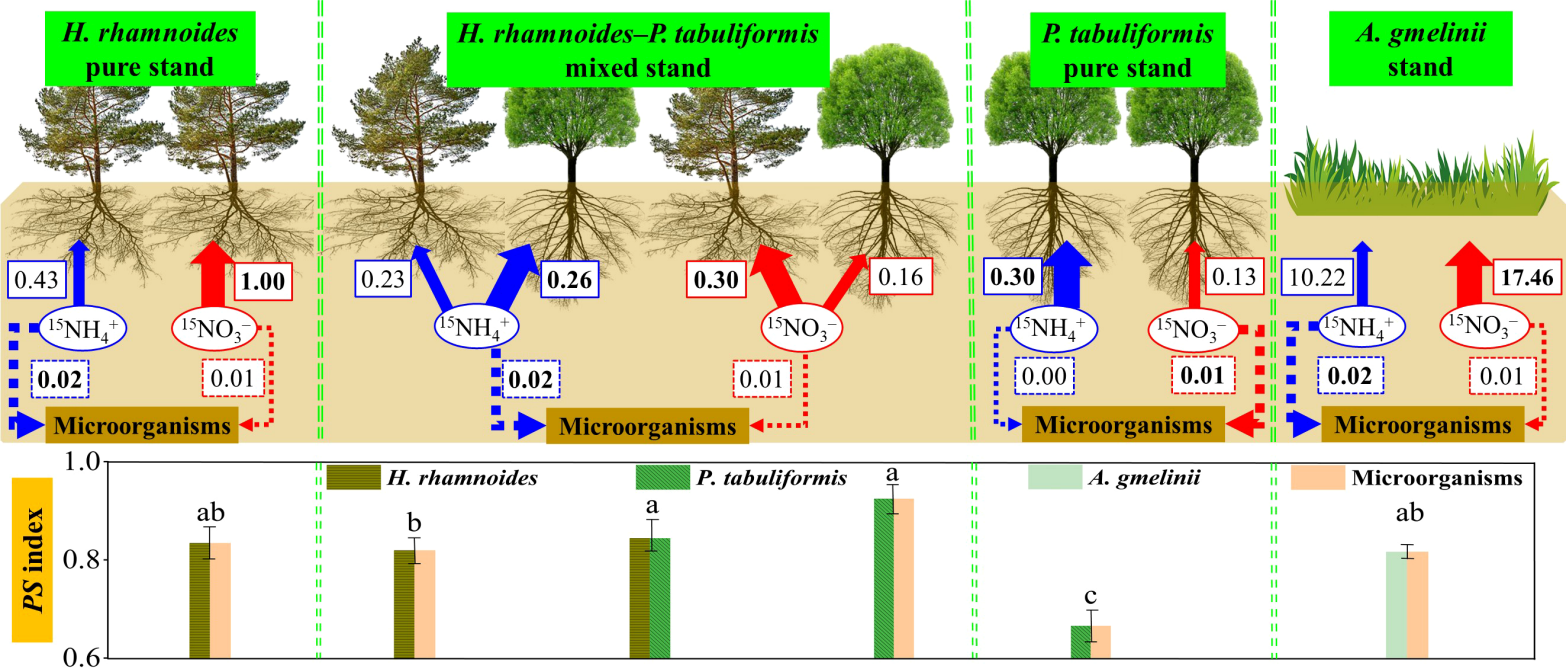
The schematic diagram of this study. The solid and dashed arrows represent the N uptake by plants and microorganisms in the four stands, respectively. The blue and red letters in the boxes represent the average uptake rates of NH_4_^+^ or NO_3_^–^ in each stand, respectively (µg ^15^N g^–1^ d^–1^). Different letters indicated significant differences of *PS* index among the four stands (P< 0.05). Bold numbers and lines represent the main forms of inorganic N preferred by plants and microorganisms.

A major aim of this study was to explore the N preferences of two dominant afforestation tree species and grass in the forest–grassland transition zone on the Loess Plateau. The results showed that *H. rhamnoides* (p) associated with ACM had a strong preference for NO_3_^–^, although NH_4_^+^ was the main N form in the soil (**Figures 2A**, **5**), which supports the first hypothesis. There may be two possible reasons for the preference for NO_3_^–^. First, soil NO_3_^–^ has a higher fluidity and solubility than NH_4_^+^, and thus, can more easily transport to the root surface (Zhu et al., 2019; Hu et al., 2021), which would promote *H. rhamnoides* uptake of NO_3_^–^. In contrast, NH_4_^+^ is easily adsorbed by microorganisms and organic matter in the soil due to enzyme production based on their needs and the availability of substrates (Geisseler et al., 2010; Zhou et al., 2020). Second, the higher uptake rates for NO_3_^–^ than NH_4_^+^ might be related to different uptake systems. NR converts NO_3_^–^ into NO_2_^–^, which is the first and rate–limiting step in nitrate assimilation (Zhou et al., 2020). However, NH_4_^+^ must be converted into an organic form by GS before it can be transported throughout the plant (Zhu et al., 2019). Thus, the higher NR activity and lower GS activity (**Table 2**) of *H. rhamnoides* (p) may have promoted the uptake of NO_3_^–^ by plants compared to that observed in the other three stands (Meng et al., 2016; Hu et al., 2021).

*Pinus tabuliformis* (p) took up more NH_4_^+^ compared to NO_3_^–^ during the 15 day experimental period (**Figures 2B**, **5**; **Supplementary Figures S7, S8B**). That finding confirmed that conifer trees were more inclined to take up NH_4_^+^ compared to NO_3_^–^ (Gruffman et al., 2014; Castro–Rodríguez et al., 2017; Gurmesa et al., 2022). ECM symbiosis may be a potential mechanism for the high NH_4_^+^ uptake rate because of the assimilation by extraradical mycelium and high-affinity NH_4_^+^ transport genes (Andersen et al., 2017; Castro–Rodríguez et al., 2017). In addition, the highest soil NH_4_^+^:NO_3_^–^ ratios in the *P. tabuliformis* pure stand among the four stands may also contribute to the uptake of NH_4_^+^ over NO_3_^–^ by *P. tabuliformis* (p) (Cui et al., 2017; Hong et al., 2018). Overall, the combination of ECM and the highest NH_4_^+^: NO_3_^–^ ratios resulted in a higher NH_4_^+^ but a lower NO_3_^–^ uptake by *P. tabuliformis* compared to *H. rhamnoides* (**Table 1**).

Inconsistent with the second hypothesis, *A. gmelinii* had a higher uptake rate of NO_3_^–^ than NH_4_^+^ (**Figures 2E**, **5**; **Supplementary Figures S7, S8C**). *A. gmelinii* has a shorter lifespan and faster growth rates, and therefore, the preference for NO_3_^–^ could be attributed to the high mobility of NO_3_^–^, which allows the N demand of *A. gmelinii* to be met (Xiong et al., 2021; Yuan et al., 2024). This conclusion is supported by the significantly higher uptake rate of NO_3_^–^ by *A. gmelinii* leaves than NH_4_^+^ (**Supplementary Figure S5**). In addition, the higher root NR activity (**Table 2**) in grassland may also promote the uptake of NO_3_^–^ by *A. gmelinii* (Meng et al., 2016; Hu et al., 2021).

The two added N forms (^15^NH_4_^+^ and ^15^NO_3_^–^) are labeled with the same molar, but due to the imbalance in soil NH_4_^+^ and NO_3_^–^ concentrations, the tracers were diluted to varying degrees (Buchmann et al., 1995). Taking the dilution effect into account, the relative proportion of NO_3_^–^ to total ^15^N tracer uptake ranged from 57.23% to 77.98% for the complete plant by *H. rhamnoides* (p) and *A. gmelinii* (at days 3, 7,15; **Supplementary Table S2**). The highest proportion of ^15^NH_4_^+^ occurred in *P. tabuliformis* (p), accounting for 75.06%, 77.93%, and 73.56% of total N uptake at days 3, 7,15 (**Supplementary Table S2**). This study also supports the conclusion of Zhu et al. (2021) that tracers with high enrichment can reduce the dilution of the inorganic soil N pool. Therefore, the dilution effect did not affect the preferences of plants for different N forms in this study.

### 4.2 Mixed afforestation did not alter the N uptake patterns by plants

Previous studies indicated that interspecific plant competition could affect the preference of N form by plants (Song et al., 2017). However, mixed afforestation did not alter the N preference by plants in this study, but decreased the N uptake rate of *H. rhamnoides*. This finding suggested that the N uptake patterns of plants mainly depended on species rather than soil N availability, which is also consistent with studies by Song et al. (2017) and Hu et al. (2021).

The strategy of niche complementarity has been proposed as a mechanism to alleviate soil N limitation and promote species coexistence (Ashton et al., 2010; Pang et al., 2019). This suggestion was supported by the results of the present study, which suggest that *H. rhamnoides* (m) and *P. tabuliformis* (m) tend to exploit different soil N forms to meet their N demands (**Figures 2C, D**, **5**). The *PS* index also confirmed that the different N preferences of plants was an effective method to alleviate N limitation in the mixed stand. Additionally, the relatively higher level of NH_4_^+^ in the soil can also meet the needs of *P. tabuliformis* and microorganism growth (**Figure 1**; **Table 1**). The decrease in the ^15^NH_4_^+^ and ^15^NO_3_^–^ uptake rate of *H. rhamnoides* may be linked to the limitation of light. Plants must allocate the C produced by photosynthesis to roots to obtain N from soil (Perkowski et al., 2021). However, the tree height and average crown diameter of *P. tabulaeformis* were larger than *H. rhamnoides*, and the average crown diameter of *P. tabulaeformis* was larger than the planted distance between them in the present study (**Table 2**). Therefore, the limitation of light by *P. tabulaeformis* led to a lower level of photosynthesis and N demand of *H. rhamnoides* in the mixed stand compared to that in the pure stand (Tang et al., 2019). Thus, *H. rhamnoides* had an insufficient C cost associated with obtaining N, resulting in a decrease in the N uptake rate. In addition, the relatively low *R_mineralization_* and *R_nitrification_* may limit N uptake of *H. rhamnoides*, which has a high N demand, compared to *P. tabulaeformis* (Van den Berg et al., 2016; Mao et al., 2021).

### 4.3 Competition for inorganic N between plants and soil microorganisms

Microorganisms preferred NH_4_^+^ over NO_3_^–^ in the pure *H. rhamnoides*, mixed, and grasslands stands, despite the dilution impact being considered (**Figures 3**, **5** **;** **Supplementary Figure S9**). This result was consistent with previous studies indicated that microorganisms preferred NH_4_^+^ due to the lower energy costs associated with NH_4_^+^ uptake (Berger et al., 2008; Fraterrigo et al., 2011; Liu et al., 2016). In addition, the relatively high NH_4_^+^:NO_3_^–^ ratio in soil may meet the N demands of microorganisms(**Figure 1**; **Table 1**), which further promotes N uptake.

The finding that microorganisms exhibited a higher uptake rate for NO_3_^–^ than for NH_4_^+^ in the *P. tabuliformis* plot was unexpected (**Figures 3B**, **5** **;** **Supplementary Figure S9B**). A possible explanation may be related to the competition between plants and microorganisms for N (Harrison et al., 2007; Andersen et al., 2017). Previous study suggested that plants and microorganisms take up different forms of N to alleviate N competition (Kaštovská and Šantrůčková, 2011). Thus, microorganisms may mainly rely on NO_3_^–^ uptake to alleviate competition with *P. tabuliformis* for NH_4_^+^ (**Figure 3B**; **Table 3**).

Prior studies also suggested that contrasting forms of N uptake between plants and microorganisms could largely result in niche complementarity (Ashton et al., 2010; Liu et al., 2022). As confirmed by this study, niche complementarity predicts that species can alleviate N competition by differing their preference for N forms (Ashton et al., 2010; Liu et al., 2020). The *PS* index between plants and microorganisms was lower by taking up different N forms than those taking up same N forms (**Table 3**). In addition, the *PS* index indicated that even plants take up different N forms, the competition between two plants tends to be higher than that between plants and microorganisms, which may be related to the duration of the experiment. Previous studies indicated that N uptake by plants significantly increased 24 h after adding tracers, while microbial N uptake was “saturated” within 24 h because of insufficient C availability (Inselsbacher et al., 2009). Meanwhile, the unidirectional N flux from soil to roots promotes N migration from microorganisms to plant roots, which enables plants to gain an advantage over microorganisms during long–term competition for N (Kaštovská and Šantrůčková, 2011; Kuzyakov and Xu, 2013). The competition between plants and soil microorganisms for inorganic N directly affects the ecosystem productivity and stability. Besides, differences in their preferences for inorganic N can enhance N available and reduce N leaching losses. Based on the spatiotemporal niche differentiation of N between plants and microorganisms, N supply can be effectively regulated. This allows for optimized fertilization and soil management strategies, which not only enhance ecosystem productivity but also contribute to sustainable land management and ecological restoration in N-limited areas.

## 5 Conclusion

This study demonstrated that the N preference of plants was influenced by different root mycorrhizal symbiosis types under similar soil N conditions. *H. rhamnoides* was associated with ACM preference for NO_3_^–^. Conversely, *P. tabuliformis*, which associated with ECM, exhibited the preference for NH_4_^+^ due to the high assimilation of NH_4_^+^ by extraradical mycelium. In addition, mixed afforestation did not alter the N preference of plants. *A. gmelinii* preferred to take up NO_3_^–^, which is highly mobile. The different N uptake forms preferred by plants and soil microorganisms resulted in a low *PS* index, indicating that niche complementarity may be a potential mechanism to decrease niche overlap and maintain community stability. Given that mycorrhiza is ubiquitous in majority plants, these findings may provide strong evidence for the importance of the mycorrhizal type in plant N uptake patterns.

## Data Availability

The original contributions presented in the study are included in the article/**Supplementary Material**. Further inquiries can be directed to the corresponding author.
