# Supplementary material for: Different nitrogen uptake patterns of plant and soil microorganisms in the forest-grassland transition zone on the Loess Plateau
Source: Front Plant Sci. 2025 Jan 21;15:1480517. doi: 10.3389/fpls.2024.1480517 (PMC11790565; doi:10.3389/fpls.2024.1480517)

Different nitrogen acquisition patterns of plant and soil microorganisms in the forest-grassland transition zone on the Loess Plateau

Lina Wang[1], Xu Deng[1], Ying Zhou[2], Xueqi Geng[2], Zeling Zhang[2], Yakun Tang[2*]

[1]College of Forestry, Northwest A&F University, Yangling, Shaanxi 712100, China; [2] State Key Laboratory of Soil Erosion and Dryland Farming on the Loess Plateau, Northwest A&F University, Yangling, Shaanxi 712100, China

E–mail addresses for corresponding author (*):

Yakun Tang, E–mail: yktang@nwafu.edu.cn; Tel: 86–29–87081910; Fax: 86–10–64858099

Figure. S1 Locations of the study region and four studied stands in the forest–grassland transition zone on the Loess Plateau, China.

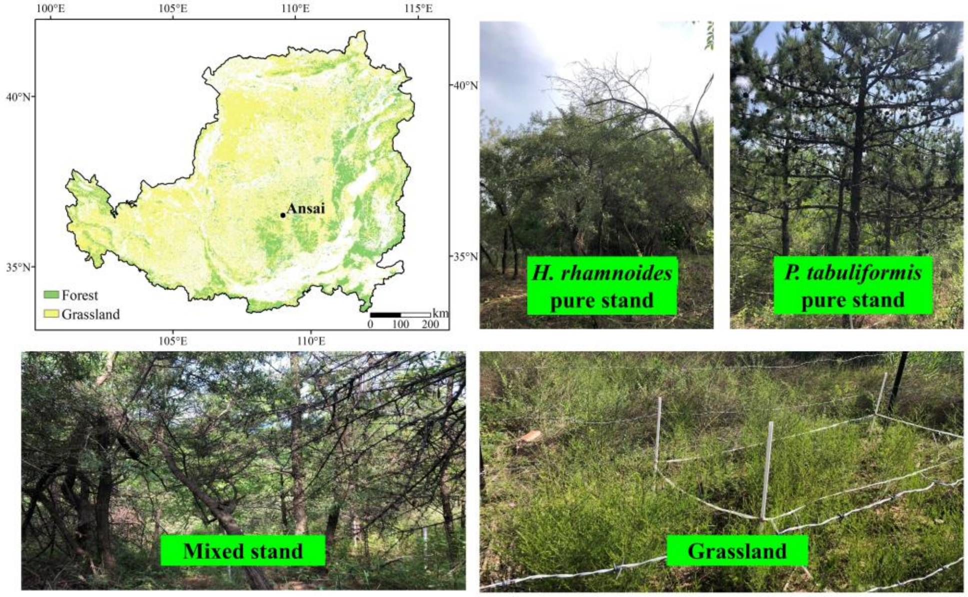

Figure. S2 Rainfall (mm) and 0–50cm soil moisture content (%) in the four studied stands before and after ^{15}N labeling. Arrows represent the rainfall and moisture content at 3, 7, and 15 days after labeling.

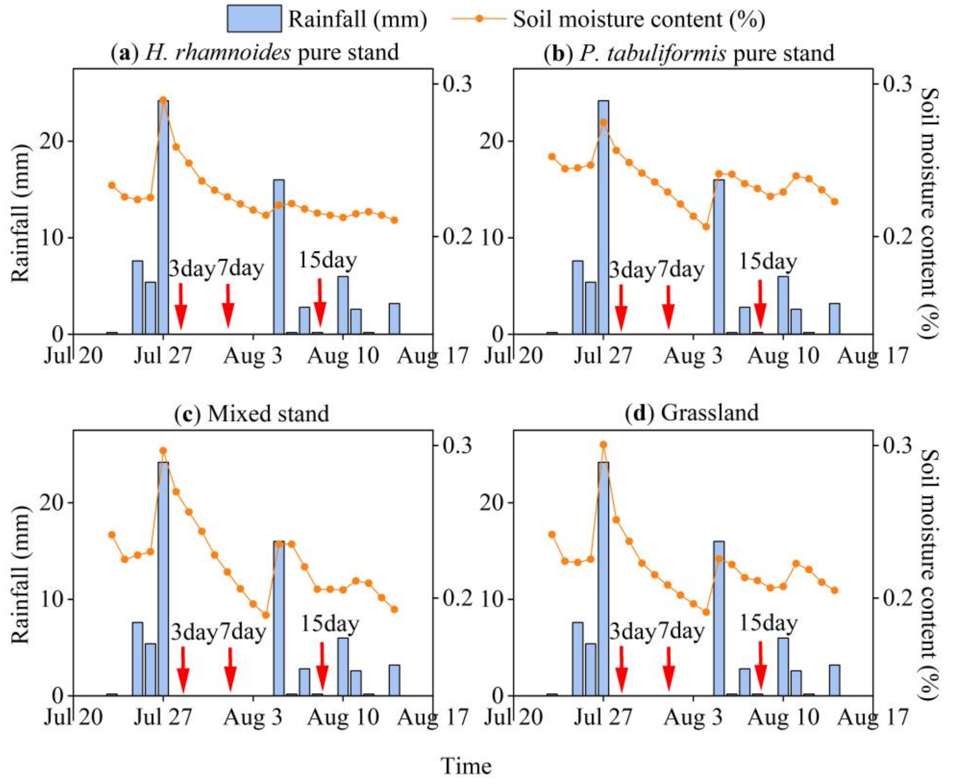

Figure. S3 The average proportion of root surface area between 0–20 cm, 20–50 cm, 50–100 cm in the range of 0–100 cm in the four studied stands (means ± SE, n = 9).

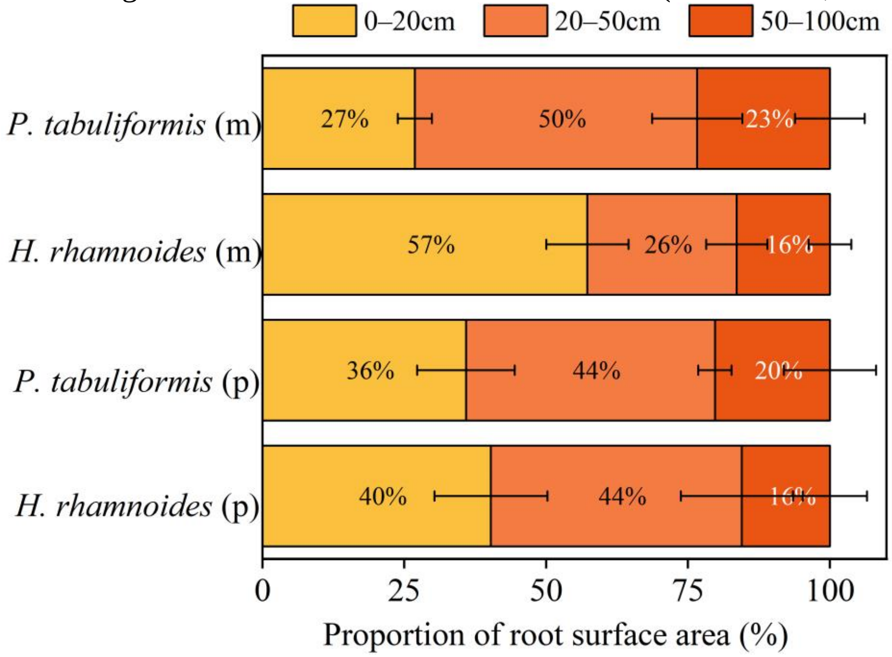

Figure. S4 The $\delta^{15}N$ of different plant organs in the tree stands of (a, b) *H. rhamnoides* and (c, d) *P. tabuliformis* (means ± SE, n = 3). Asterisks indicate significant differences in the ^{15}N enrichment between N forms within each stand (ANOVA and repeated measurements. *, $P < 0.05$; **, $P < 0.01$). D: Time since ^{15}N labeling (day); L: The form of applied ^{15}N–labeled N. (p) and (m) represent pure and mixed stands, respectively.

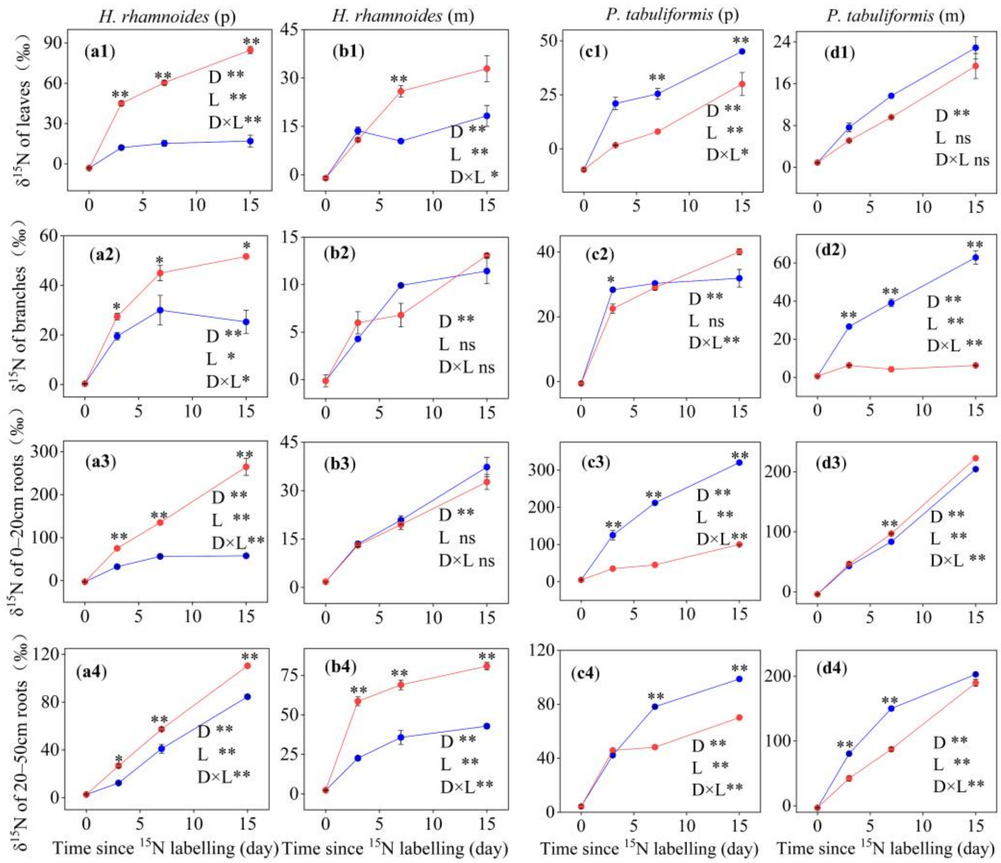

Figure. S5 Uptake rates of ^{15}N tracer of leaves, branches, fine roots collected from 0–20 cm, 20–20 cm, and 0–50 cm soil in the tree stands of (a, b) *H. rhamnoides* and (c, d) *P. tabuliformis* (means ± SE, n = 3). Asterisks indicate significant differences in the ^{15}N uptake rates between N forms within each stand (ANOVA and repeated measurements. *, $P < 0.05$; **, $P < 0.01$). D: Time since ^{15}N labeling (day); L: The form of applied ^{15}N–labeled N. (p) and (m) represent pure and mixed stands, respectively.

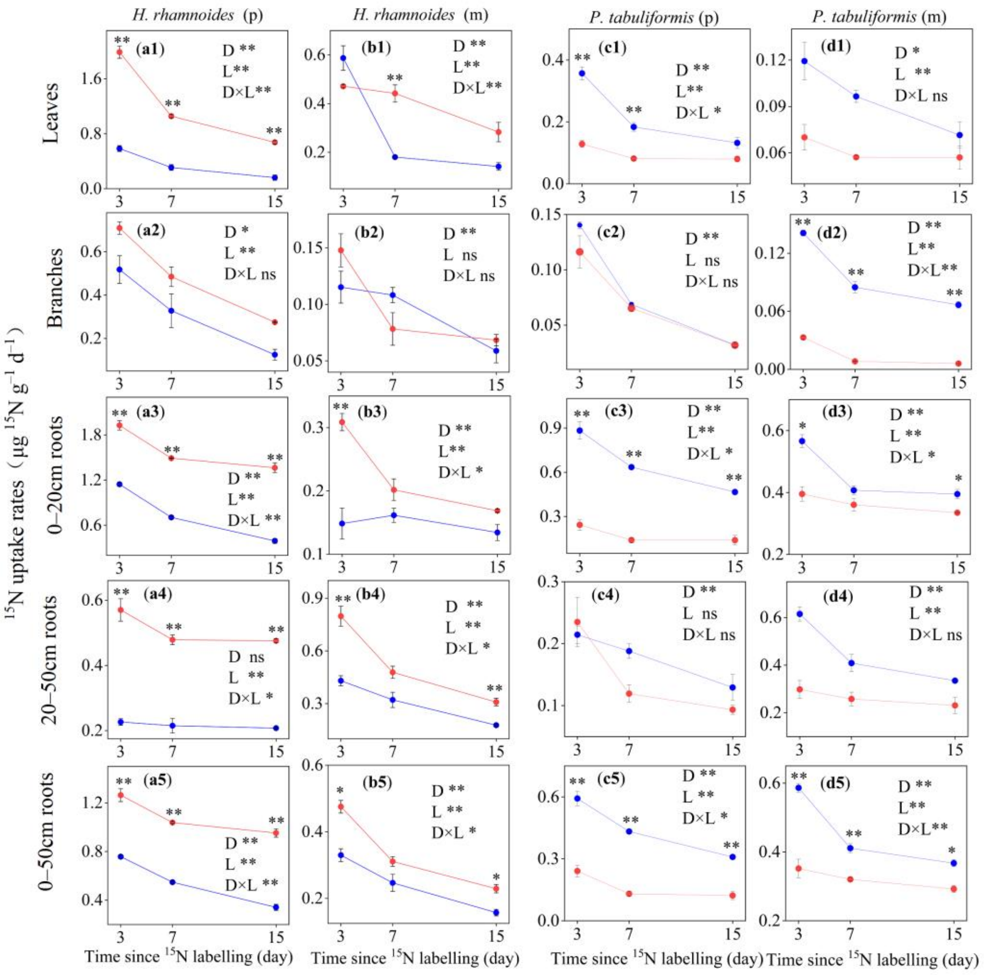

Figure. S6 The $\delta^{15}N$ and uptake rates of ^{15}N tracer of leaves (a, c) and fine roots (b, d) in *A. gmelinii* stand (means ± SE, n = 3). Asterisks indicate significant differences in the ^{15}N uptake rates between N forms within each stand (ANOVA and repeated measurements. *, $P < 0.05$; **, $P < 0.01$). D: Time since ^{15}N labeling (day); L: The form of applied ^{15}N–labeled N.

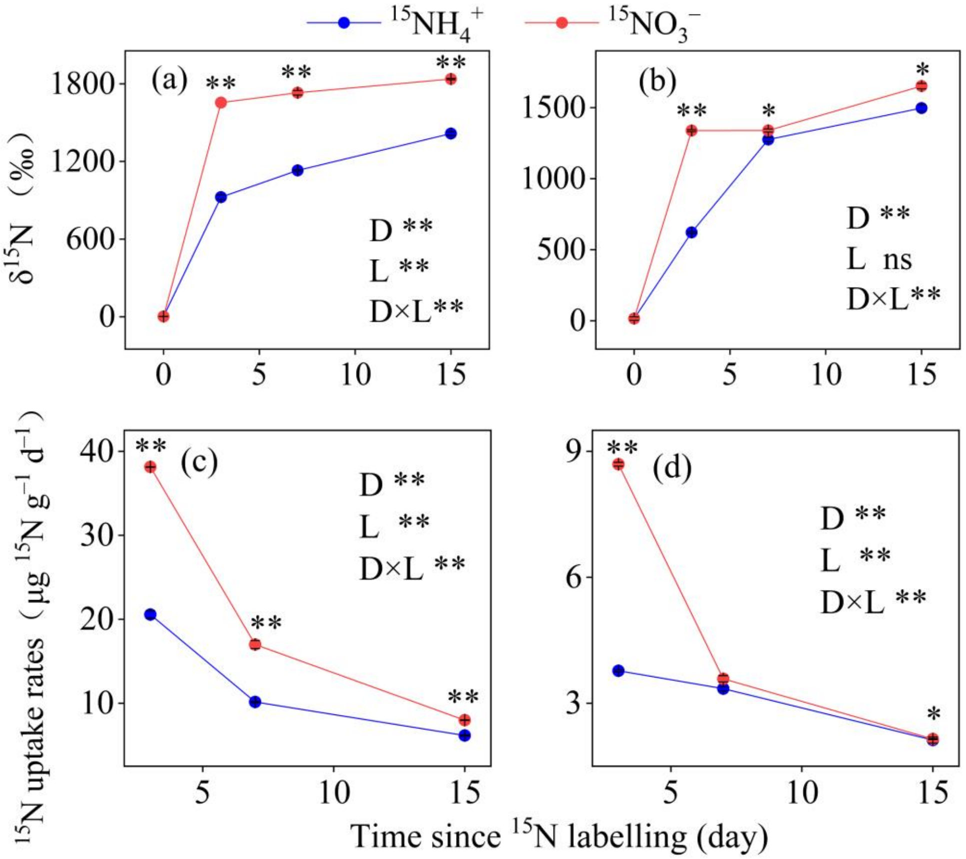

Figure. S7 The relative proportion of $^{15}NH_4^+$ and $^{15}NO_3^-$ to total ^{15}N tracer uptake by the whole plant in the four stands of (a, d) *H. rhamnoides*, (b, e) *P. tabuliformis* and (c) *A. gmelinii* at 3, 7 and 15 days after ^{15}N labeling, respectively (shown are N–pool–weighted mean by roots, leaves and branches, n = 3). (p) and (m) represent pure and mixed stands, respectively.

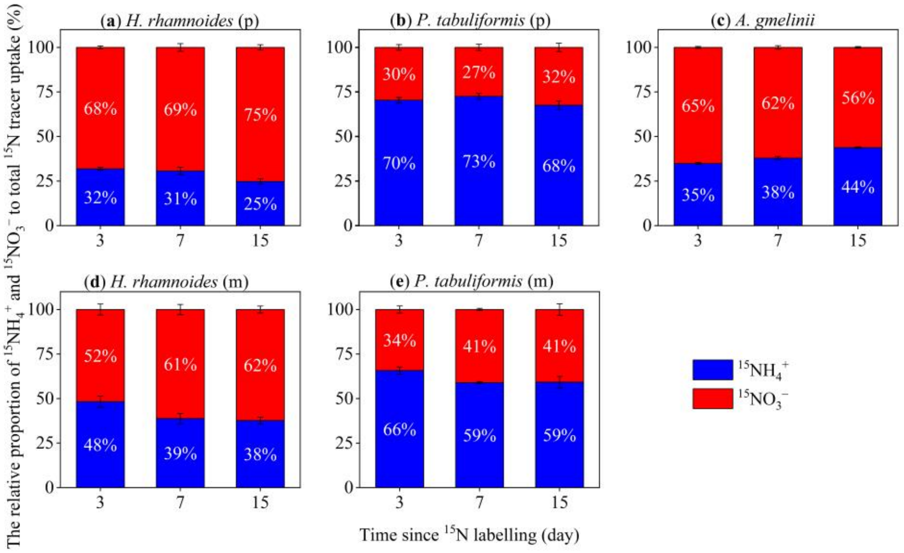

Figure. S8 The relative proportion of $^{15}NH_4^+$ and $^{15}NO_3^-$ to total ^{15}N tracer uptake by different plant organs (roots, leaves and branches) and whole plant (N–pool–weighted mean) in four studied stands of (a, d) *H. rhamnoides*, (b, e) *P. tabuliformis* and (c) *A. gmelinii* at 3, 7 and 15 days after ^{15}N labeling (n = 3). (p) and (m) represent pure and mixed stands, respectively.

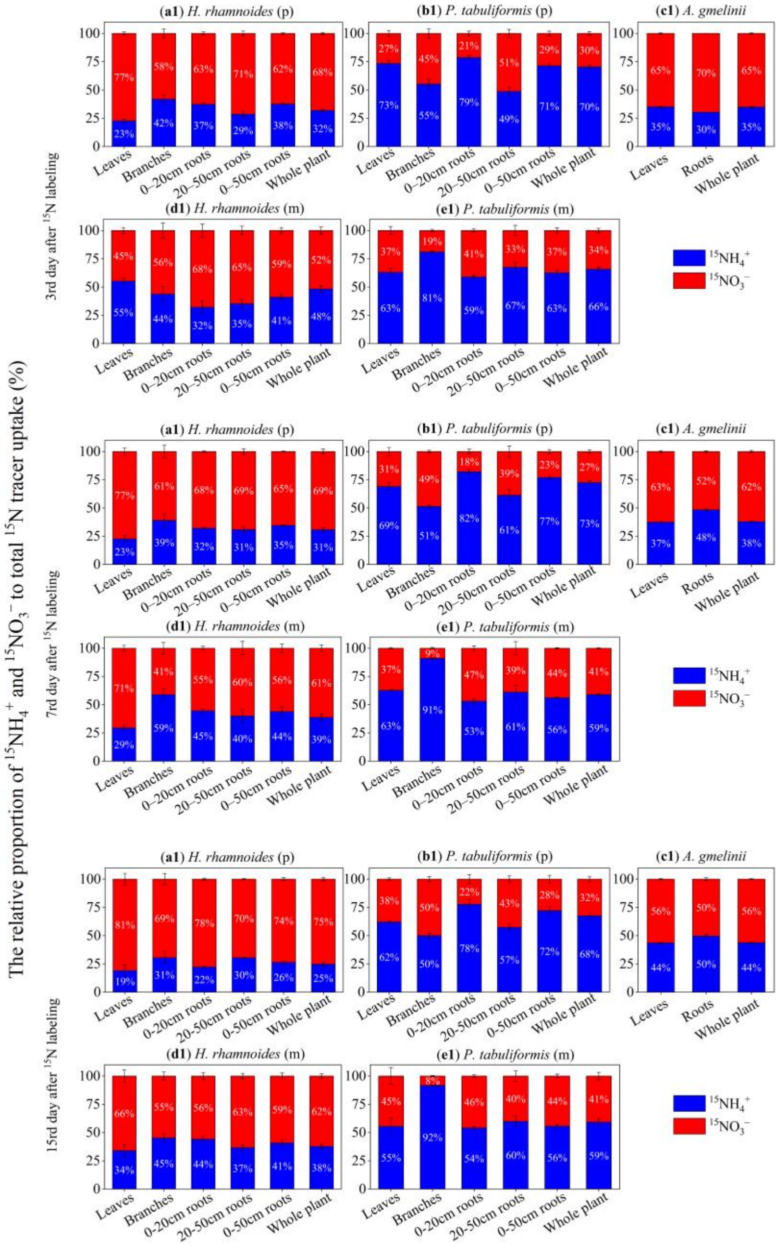

Figure. S9 The relative proportion of $^{15}NH_4^+$ and $^{15}NO_3^-$ to total ^{15}N tracer uptake by soil microorganisms in the four stands at 3, 7 and 15 days after ^{15}N labeling, respectively (n = 3).

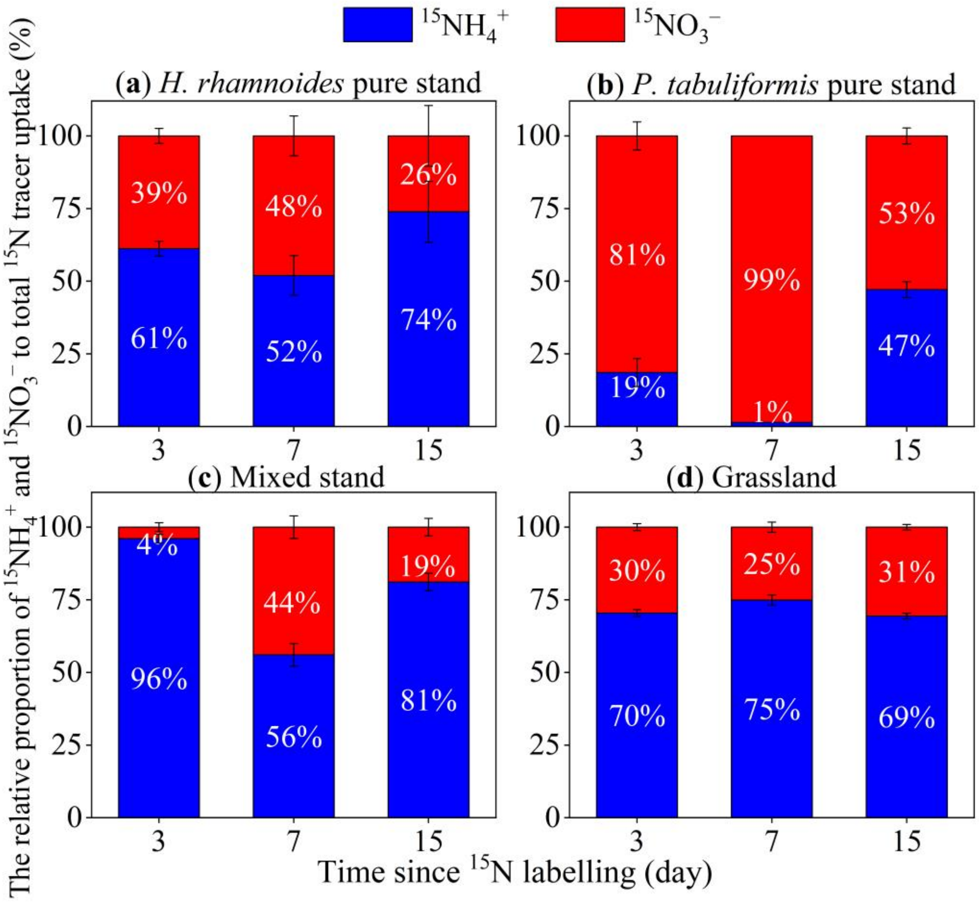

Supplement: Supplementary file 1 [file DataSheet1.pdf]
