# Supplementary material for: Different nitrogen uptake patterns of plant and soil microorganisms in the forest-grassland transition zone on the Loess Plateau
Source: Front Plant Sci. 2025 Jan 21;15:1480517. doi: 10.3389/fpls.2024.1480517 (PMC11790565; doi:10.3389/fpls.2024.1480517)
Supplement: Supplementary file 2 [file DataSheet2.pdf]

Different nitrogen acquisition patterns of plant and soil microorganisms in the forest-grassland transition zone on the Loess Plateau

Lina Wang[1], Xu Deng[1], Ying Zhou[2], Xueqi Geng[2], Zeling Zhang[2], Yakun Tang[2*]

[1]College of Forestry, Northwest A&F University, Yangling, Shaanxi 712100, China; [2] State Key Laboratory of Soil Erosion and Dryland Farming on the Loess Plateau, Northwest A&F University, Yangling, Shaanxi 712100, China

E–mail addresses for corresponding author (*):

Yakun Tang, E–mail: yktang@nwafu.edu.cn; Tel: 86–29–87081910; Fax: 86–10–64858099

Table S1 *F*–statistics of one–way ANOVA of the effects of applied N form ($^{15}NH_4^+$, $^{15}NO_3^-$) on soil NH_4^+, NO_3^-, inorganic N, and total N concentrations in the four studied stands (n = 3).

Soil layer (cm)	N concentration (mg/L)	*H. rhamnoides* pure stand		*P. tabuliformis* pure stand		Mixed stand		Grassland	
		F	P	F	P	F	P	F	P
0–20	NO_3^-	1.581	0.231	1.956	0.159	1.121	0.368	1.607	0.225
	NH_4^+	2.868	0.067	0.676	0.578	2.303	0.114	1.251	0.322
	$NO_3^- + NH_4^+$	2.890	0.066	2.227	0.122	1.415	0.273	1.213	0.335
20–50	NO_3^-	1.052	0.395	1.056	0.394	1.176	0.348	1.880	0.171
	NH_4^+	2.244	0.120	0.787	0.518	1.024	0.407	0.819	0.501
	$NO_3^- + NH_4^+$	2.820	0.070	0.734	0.546	0.845	0.488	0.609	0.618

Table S2 Taking the dilution effect into account, the relative proportion of $^{15}NH_4^+$ and $^{15}NO_3^-$ to total ^{15}N tracer uptake by the whole plant and microorganisms in the four stands at 3, 7, and 15 days after ^{15}N labeling, respectively (shown are N–pool–weighted mean by roots, leaves, and branches, n = 3).

Stands	Object	3		7		15	
		$^{15}NH_4^+$	$^{15}NO_3^-$	$^{15}NH_4^+$	$^{15}NO_3^-$	$^{15}NH_4^+$	$^{15}NO_3^-$
H. rhamnoides	*H. rhamnoides*	28.60(0.80)	71.40(0.80)	27.47(2.00)	72.53(2.00)	22.02(1.36)	77.98(1.36)
pure stand	Microorganisms	57.45(3.24)	42.55(3.24)	48.32(8.44)	51.68(8.44)	83.05(7.30)	16.95(7.30)
P. tabuliformis	*P. tabuliformis*	76.06(1.35)	23.94(1.35)	77.93(1.49)	22.07(1.49)	73.56(2.09)	26.44(2.09)
pure stand	Microorganisms	23.10(7.10)	76.90(7.10)	47.08(2.31)	52.92(2.31)	59.07(10.42)	40.93(10.42)
	H. rhamnoides	27.23(1.72)	72.77(1.72)	33.32(0.59)	66.68(0.59)	33.13(2.93)	66.87(2.93)
Mixed stand	*P. tabuliformis*	43.54(3.09)	56.46(3.09)	53.18(2.98)	46.82(2.98)	54.33(1.99)	45.67(1.99)
	Microorganisms	98.87(4.95)	1.13(4.95)	47.90(4.75)	52.10(4.75)	75.70(4.44)	24.30(4.44)
Grassland	*A. gmelinii*	33.93(0.54)	66.07(0.54)	36.96(0.97)	63.04(0.97)	42.77(0.45)	57.23(0.45)
	Microorganisms	69.62(1.47)	30.38(1.47)	74.16(2.17)	25.84(2.17)	68.58(1.19)	31.42(1.19)

Each Value is presented as mean value with SE in parentheses.
